# Changes in Clinical Training for Nursing Students during the COVID-19 Pandemic: A Scoping Review

**DOI:** 10.3390/nursrep13010035

**Published:** 2023-03-01

**Authors:** Catarina Lobão, Adriana Coelho, Vitor Parola, Hugo Neves, Joana Pereira Sousa, Rui Gonçalves

**Affiliations:** 1 Health Sciences Research Unit: Nursing (UICISA: E), Nursing School of Coimbra (ESEnfC), 3000 Coimbra, Portugal; 2 Portugal Centre for Evidence-Based Practice: A Joanna Briggs Institute Centre of Excellence (PCEBP), 3000 Coimbra, Portugal; 3 School of Health Sciences—Polytechnic of Leiria, Center for Innovative Care and Health Technology—ciTechCare, 2411-901 Leiria, Portugal

**Keywords:** changes, clinical training, COVID-19, nursing students, review

## Abstract

(1) Background: The COVID-19 pandemic has cost social, economic, cultural, and educational life, distressing nursing training and practice. This study aimed to map the literature on changes in clinical training for nursing students during the COVID-19 pandemic. (2) Methods: A scoping review was conducted according to JBI methodology’s latest guidance. A set of relevant electronic databases and grey literature was searched to report results published in English, Spanish, and Portuguese. (3) Results: A total of 12 studies were included in the study, addressing changes in clinical training in undergraduate nursing students due to COVID-19 pandemic activity, published between 2020 and 2022. (4) Conclusions: Nursing schools made an effort to replace traditional clinical training with several activities, primarily based on simulation or virtual activities. However, contact with others is essential, and simulation programs or scenarios cannot provide it.

## 1. Introduction

The emergence and effect of the SARS-CoV-2 coronavirus transformed educational approaches, as clinical settings were no longer available for internship, and nursing schools had to replace and reshape clinical scenarios [1] by reinventing strategies and adjusting teaching, learning, and assessment methods in nursing education [2,3].

It created unprecedented opportunities for nursing education as it required creative teaching techniques to promote students’ clinical learning, ensuring that the necessary learning outcomes and professional competencies were achieved [4,5].

The traditional clinical practice and face-to-face experiences were replaced by technological environments, for both students and faculty, with screen-based simulation [4], remote or virtual simulated learning experiences using commercial products or telehealth [6], and technology-enhanced storyboard techniques [7]. Moreover, nursing schools were unprepared for remote instruction transition during the COVID-19 pandemic, which challenged their curricula [5].

The discipline of nursing focuses on human reactions to health disease occurrences and life processes, where face-to-face nursing care is vital [8]. Thus, training students who will be qualified nurses caring for people involves developing specific skills, reflecting on role-playing discussions, exchanging clinical experiences, professional and multidiscipline relationships, and critical thinking [8].

The pandemic raised numerous challenges in teaching nursing students, specifically in the clinical context. This new reality allowed [9] students to achieve the required clinical hours and therefore complete their degrees if they were senior students. On the other hand, young nursing students had their clinical placements delayed due to rapid changes in the clinical environment. Conversely, lock-in policies forced junior students to discontinue or delay clinical education [9]. What alternatives were offered to these students?

According to JBI methodology and the previously published review protocol [10], we conducted a scoping review to map the changes in clinical training for undergraduate nursing students during the COVID-19 pandemic.

Moreover, it would be relevant to do this mapping at any level of education. The focus on undergraduate students is because the fundamentals of nursing are acquired in this period. During training, when undergraduate students receive the information and abilities that set nurses apart from laypeople as professional healthcare providers, it is a crucial time in the professional development of nursing students [11]. If it is compromised, the repercussions will manifest from the base of the nursing profession.

This review aims to understand how faculties adapted curricula to face the problem of inaccessibility to clinical settings and how academics developed programs to target clinical teaching, learning, and assessment strategies for nursing students in similar contexts. This map identified relevant topics on nursing education strategies to improve nursing students’ knowledge development and helped identify potential research gaps. This mapping will support, in the near future, comparison studies between changes in teaching before and after the pandemic, and comparison studies between changes implemented temporarily and those which “came to stay”.

An initial search of MEDLINE (PubMed), the J.B.I. Evidence Synthesis, the Cochrane Database of Systematic Reviews, PROSPERO, and Open Science Framework (O.S.F.) revealed that, currently, there are no scoping reviews or systematic reviews (published or in progress) about this subject [12,13,14].

This scoping review was developed to answer the following questions:

- What are the changes in clinical practice training for nursing students during the COVID-19 pandemic? (By change it means an alternative to clinical practice in context).
- What is the context of clinical practice training for nursing students where the changes are described? (By context it means the level/year of training).
- What are the academic and personal implications in the nursing student learning process? (By implications, it is intended to map the consequences of the training changes on a personal or academic level).

## 2. Materials and Methods

The JBI latest guidance methodology guided this scoping review [12,13,14], and was reported following the Preferred Reporting Items for Systematic Reviews and Meta-Analyses Extension for Scoping Reviews (PRISMA-ScR) guidelines [15]. This review protocol has been previously published [10].

### 2.1. Inclusion Criteria

According to the JBI recommendations mnemonic “P.C.C.” for scoping reviews, the inclusion criteria were: **Participants—**Undergraduate nursing students. **Concept—**Studies exploring nursing students’ clinical training changes during the COVID-19 pandemic. **Context—**Any clinical practice setting, independent of the country of the study. **Types of sources—**Studies with quantitative, qualitative, and mixed methods study design. In addition to these, all types of systematic review were considered.

### 2.2. Search Strategy

The search strategy was used to identify published and unpublished primary studies and reviews.

Two reviewers developed the search strategy and peer-review by an expert third reviewer who considered the Peer Review of Electronic Search Strategies (PRESS) checklist [16]. The JBI recommended that three-step search strategy was applied [12,14]. Records in English, Spanish, and Portuguese were included to ensure a suitable selection procedure and data extraction.

The search strategy was adapted to the specificities of each information source. The databases searched included MEDLINE (via PubMed); CINAHL Complete (EBSCOhost); Cochrane Central Register of Controlled Trials; Cochrane Database of Systematic Reviews; LILACS; Scopus; and SciELO. The search for unpublished studies included DART-Europe and OpenGrey. As an example, the search strategy used for MEDLINE (via PubMed) is presented in Table 1. The search was structured in both Medical Subject Headings (MeSH) and text words shown in the literature. The terms were combined using truncation symbols and Boolean operators (“OR” and “AND”). Lastly, the reference lists of the articles included in the review were screened for supplementary papers.

Study languages were restricted to those mastered by the authors—English, Spanish, and Portuguese—to ensure a good-quality selection procedure and data extraction. No time limit was considered in this review. However, the research has considered the COVID-19 pandemic; as such, all the studies included were dated equal to or greater than 2019.

Furthermore, since this scoping review aims to map the changes in clinical training for undergraduate nursing students during the COVID-19 pandemic, no rating of the methodological quality was provided, according to the JBI methodology, and as a result, practice recommendations were provided with caution. As mentioned by JBI “no assessment of methodological quality and formal synthesis takes place as part of a scoping review” [12].

### 2.3. Study Selection and Screening Process

All the records identified over database searching were retrieved and kept in Mendeley^®^ V1.19.4 (Mendeley Ltd., Elsevier, Amsterdam, The Netherlands) and duplicates were removed. Two reviewers independently screened the titles and abstracts. A pilot test was made to verify whether inclusion criteria were met. The two independent reviewers assessed the full text of selected citations in detail against the inclusion criteria. The references of the included studies in the review were hand-searched. Disagreements among the two reviewers were solved through discussion or with a third reviewer. In the case of the inaccessible full article, the author was contacted [15].

## 3. Results

The data total of two hundred and fifty-nine studies were identified from the databases. After removing eighty-three duplicates, one hundred and seventy-six references remained. The titles and abstracts of these articles were reviewed, resulting in a total of sixty-six eligible records. After the complete reading of these records and application of the previously defined inclusion criteria, two were excluded because they were in a language different from those the research team spoke. Additionally, nine did not comply with the requirements referring to the population, six did not comply with the selected context, and thirty-seven were excluded due to the concept not being stipulated by the inclusion criteria.

**Figure 1:**
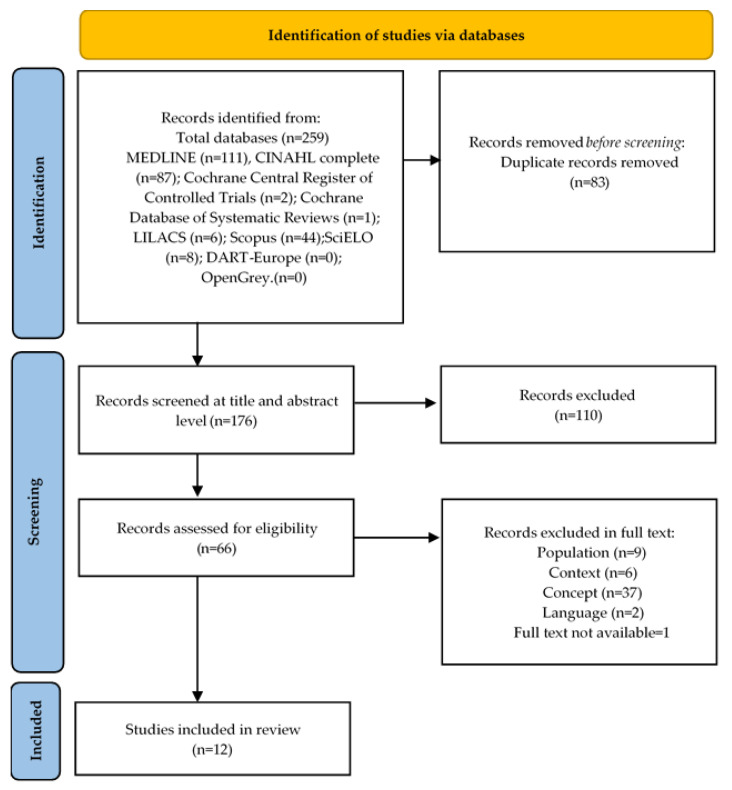
PRISMA Flowchart.

As such, after the identification and screening phases of the review procedure (Figure 1), 12 studies were included in this review.

### Data Analysis and Presentation

The characteristics of the included studies and the answers to the review question are summarised in Table 2. Of the twelve studies included in this review, one was conducted in Indonesia, six in the USA, one in Germany, one in Colombia, one in Hong Kong, and two in the Republic of Korea. The studies were published in the years 2020 (*n* = 2), 2021 (*n* = 8), and 2022 (*n* = 2).

The first study analysed was conducted by Anggraini, Chrisnawati, and Warjiman (2022) [17] in Indonesia, targeting 30 nursing students during clinical nursing training. To continue the training of those students, they used a simulation program that proved beneficial in reviewing the theories obtained previously (Table 2).

The study by Banjo-Ogunnowo and Chisholm (2022) [18] was conducted on nursing students who were developing their learning in the maternal–pediatric course. They used virtual learning as an alternative to clinical practice in hospital settings (Table 2).

The third study [19] analyzed was developed by a team of researchers from Germany. In this study, nursing students received the interprofessional COVID-19 substitution program (I-reCovEr) during clinical teaching in a pediatric setting (Table 2).

Another study included [20] was conducted in Columbia (U.S.A.) by Bradford et al. (2021) with midwifery and women’s health nurse practitioner students. Synchronous and asynchronous simulation sessions were offered for their formative learning. These simulation opportunities served as valuable adjuncts to traditional learning and provided a level of experiences to students with unequal access and capability to engage in the clinical setting (Table 2).

In the study developed by Cowperthwait et al. (2021) [21], during clinical training in psychiatric mental health nursing, eighty senior undergraduate students were allocated to a simulation that replaced the physical clinical context. The main benefits emphasized by the students were the reflection developed during the simulation sessions, the opportunity to receive feedback, and the learning acquired through the observation of other colleagues in the interaction with the same patients (Table 2).

Fung et al. (2021) [22] conducted a study in Hong Kong with 188 final-year undergraduate nursing students. In this study, a virtual simulation educational program replaced traditional clinical practice in medical and surgical cases with debriefing. In the students’ perception, this educational program was beneficial in developing clinical competence and the nursing process. However, communication and critical thinking were better applied in the traditional clinical setting (Table 2).

In a study by Hassler et al. (2021) [23], flipped clinical practice was analysed through a synchronized remote clinical experience in one clinical specialty chosen by 98 s-year nursing students. Students emphasized that they saw the methodology as successful to reinforce clinical concepts to simulate the experiences of the traditional hospital setting’s clinical training (Table 2).

Another study included in this scoping review and developed by Hwang and Chun (2021) [24] put into evidence the use of clinical practice education with virtual reality in the Republic of Korea (Table 2). Fifty-nine nursing students were divided into two groups. In this study, the experimental group was exposed to the vSim nursing program as an alternative practice to the traditional clinical practice using virtual reality. Their results showed positive benefits in clinical thinking and clinical practice performance but without wide statistical significance.

From the Republic of Korea arrived the study of Kim, Kang, and Gagne (2021) [25], which highlighted the use of a six step virtual simulation alternative program to the traditional nursing clinical practice (Table 2). The proposed six step virtual simulation alternative program evidenced the difficulties perceived by the students in using a non-native language and the impact of the specific cultural differences shown in the scenarios. On the other hand, the developed confidence and competence in providing patient-centered care were shown as benefits of virtual simulation.

The study by Revell et al. (2022) [26] disclosed the results of supplementing the traditional clinical period with an 18-h simulated experiences pack (Table 2). The authors revealed the evidence of transformative learning expressed by students. The sample of undergraduate nursing students demonstrated an evident response to the change and challenges, discovering and developing other professional competencies and skills.

In 2020, Shea and Rovera [27] developed a study with two hundred and forty-four nursing students exposed to virtual and remote simulations as telehealth with standardized patients as an alternative of half of the clinical practice hours (Table 2). During the health emergency period and the university campus closure, every effort was needed to replace clinical practice hours and stop the interruption of the nursing graduation process with simulation activities in different clinical areas.

The last analyzed study, developed by Wands, Geller, and Hallman (2020) [28], presents to the scientific community a four-week simulation program with forty-two nursing students to substitute their in-person clinical experiences (Table 2). By using four free online simulation programs, logged over 1200 h, the students referred to experiencing positive growth in different professional competencies and skills despite difficulties when trying to manage multiple devices to access the virtual sessions and materials.

Our findings show that nursing schools made an effort to replace traditional clinical training with several activities, primarily based on simulation or virtual activities, allowing students to improve their abilities in caretaking [17,18,21,24,25,27,28]. Simulation sessions were structured in steps, with suggested reading, pre- and post-simulation quizzes, interactive clinical scenarios, and reflection [25,26]. They improved communication skills by role-playing, gaining experience in practical activities, and flipping clinical practice to replicate traditional care [17,20,23]. Scholarly journals were also proposed to enhance reflection and knowledge acquisition by virtual clinical practice [26].

After simulation sessions, debriefing moments were taken where simulation and case management were analyzed [18,22] or replaced by online seminars [26].

One of the included articles, Banjo-Ogunnowo and Chisholm (2022) [18], mention as a strategy the use of a virtual platform—the i-Human platform was widely used to assess case scenarios, including patient history, physical assessment, defining nursing diagnoses, and prioritizing interventions [18,29], although other virtual platforms were used by universities [30,31].

The primary contexts varied from maternal–pediatric [18,19,23,27], women’s health [20], psychiatric mental health [21,23,27], medical–surgical health [22,26,27], adult health [23], nursing fundamentals/profession stage [17,27], and community health promotion and wellness reproductive health [27].

## 4. Discussion

In this scoping review, we identified twelve primary studies, mainly from the U.S.A., addressing changes in clinical training in undergraduate nursing students due to COVID-19 pandemic activity, published between 2020 and 2022.

Concerning the simulation time, the included articles varied in context and ranged from 18 h to 8 weeks of rotation [18,19,26,28].

The adoption of virtual lessons allowed each nursing school to define clinical training replacement time, letting students progress at their graduation level. At the end of the program, students reported that this learning methodology enabled them to continue clinical training, with advantages in reviewing concepts, nursing theories, and applying them in simulation scenarios or later engaging in clinical settings [17,20]. Reflective and debriefing periods were viewed as positive. Professor–student and student–student interaction encouraged discussion, feedback, and interchange of opinions [21,22], analysis of clinical competencies and information to include in the nursing process [22,24], and clinical concepts reinforcement [23]. A positive modification in students’ attitudes was also noticed, such as confidence, resilience, gratitude, or embracing advocacy [20,25,26,28]. On the other hand, managing multiple technological devices for videos or website material was more challenging. Additionally, simulation widgets in the English language were hard to understand for those whose native language was not English and simulations were not adapted to cultural users’ differences [25]. The lack of understanding of simulation widgets in English among non-native speakers could be attributed to language barriers, where individuals may not have a good command of the English language, making it challenging for them to understand technical terms and concepts. Additionally, cultural differences could play a role, as certain phrases or expressions may not be familiar to individuals from different cultures. To address these challenges, it may be necessary to provide language and cultural adaptations to simulation widgets. As technology advances, the need for technical skills and understanding will likely increase, making it even more critical to bridging language and cultural barriers.

The Pandemic made it challenging for nursing schools to adapt their curricula to allow students to continue their practice and advance at their graduation level. Each school sets a different program, adjusting to its needs, making its comparison difficult. Overall, synchronous or hybrid virtual classes narrowed relations between professors and students. Narrowed relations refer to the potentially reduced level of interaction and engagement between professors and students in virtual or hybrid learning environments compared to face-to-face classes. In a virtual or hybrid setting, students may feel more disconnected from their professors and peers, which can decrease the quality of interaction, collaboration, and feedback. Depending on each context, setting specific scenarios allowed a deeper reflection on practice, connecting concepts and theories. However, users also had to invest time in acquiring technological competencies, which could be time-consuming and challenge the learning process. The interaction between users and simulation programs was centered on pressing buttons rather than the natural interaction between carer and cared [25].

The results were similar when comparing clinical practice education with virtual reality and traditional learning. There were no observed differences between these two learning approaches [18,19,24], although both improved learning abilities, mainly on previous practice before clinical training, in conjunction with reflection on person-centered needs, developing communication skills, and performing decision-making in a controlled environment [17,20].

The simulation was not new in nursing, where specific practices were already used, such as resuscitation or technical training before clinical practice [32]. The pandemic set a new view for the patient through the usage of technological widgets. Clinical practice was replaced by virtual scenarios, in which the interaction between participants (students and professors) promoted a richness of sharing.

A potential limitation of this scoping review was that only studies published in English, Portuguese, and Spanish were included. Articles published in other languages may potentially add information to this review’s results. Furthermore, since the objective of this scoping review was to map, no rating of the methodological quality was used. In contrast to systematic reviews where implications or recommendations for practice are a key feature, scoping reviews are not designed to underpin clinical practice decisions; therefore, the assessment of methodological quality or risk of bias of included studies (which is critical when reporting effect size estimates) does not occur [33].

Finally, the concept “changes” was not included in the search strategy in order not to exclude potential studies relevant to the present review.

## 5. Conclusions

The impact of the coronavirus pandemic created the need to reinvent strategies and readjust teaching, learning, and assessment processes in nursing education, namely in a clinical context. This scoping review identified twelve primary studies about changes in clinical training for nursing students during the COVID-19 pandemic published between 2020 and 2022. This mapping shows that the pandemic made it challenging for nursing schools to adapt their curricula to allow students to continue their practice and advance at their graduation level.

In this sense, nursing schools tried to replace traditional clinical training with several activities based on simulation or virtual activities. However, contact with others is essential, and simulation programs or scenarios cannot provide it. Simulation is essential for skill development, however, developing technical and non-technical skills simultaneously requires direct contact with patients.

More studies should be carried out within the scope of the long-term consequences of adopting these methodologies in nursing practice.

## Figures and Tables

**Table 1:** Search strategy for MEDLINE (via Pubmed) conducted on 28 March 2022.

Search	Query	Record Retrieved
#1	“students, nursing”[MeSH Terms] OR (“nurs*”[All Fields] AND “student*”[Title/Abstract])	59,061
#2	“clinical training”[Title/Abstract] OR “clinical learning”[Title/Abstract] OR “clinical placement”[Title/Abstract] OR “clinical practice”[Title/Abstract] OR “preceptorship”[MeSH Terms]	232,745
#3	“covid 19”[MeSH Terms] OR “SARS-CoV-2”[MeSH Terms] OR “covid*”[Title/Abstract] OR “SARS-CoV-2”[Title/Abstract]	237,488
#4	(“students, nursing”[MeSH Terms] OR (“nurs*”[All Fields] AND “student*”[Title/Abstract])) AND (“clinical training”[Title/Abstract] OR “clinical learning”[Title/Abstract] OR “clinical placement”[Title/Abstract] OR “clinical practice”[Title/Abstract] OR “preceptorship”[MeSH Terms]) AND (“covid 19”[MeSH Terms] OR “SARS-CoV-2”[MeSH Terms] OR “covid*”[Title/Abstract] OR “SARS-CoV-2”[Title/Abstract])	111

**Table 2:** Articles included in the scoping review.

Author Year Country	Population	Changes in Clinical Training	Context of Clinical Training	Implications
Anggraini, S., Chrisnawati, C. & Warjiman, W., 2022 Indonésia [17]	30 nursing students	Applying the Hospital Clinical Practice Based Simulation (HCPBS) Model to the practical learning outcomes of nursing profession students. This model provides a practical experience close to hospital conditions in which professional nursing students gain experience in caring for patients, communicating with patients and families in role-play, and case management.	Nursing Profession stage	It was effective in increasing the practical learning achievement of nursing profession students. It was beneficial to review the theories that had been obtained previously. They have carried practice out in the form of practice both with friends and with phantoms so that they can still apply their expertise/skills according to theory. An effective learning strategy cannot replace real life but must be used as an addition to the learning process.
Banjo-Ogunnowo, S. & Chisholm, L., 2022 USA [18]	Nursing students (Licensed Vocational Nurses (LVN) to Associate Degree Nursing (ADN) students)	Uses virtual learning as an alternative to in-hospital clinic. Group 1 (traditional learning): students participated in four-hour classroom lectures, two 2-h labs, and one 12-h clinical experience per week for 8 weeks during the Spring 2019 semester. Group 2 (virtual learning): students participated in 4-h virtual lectures, two 2-h virtual lab sessions, and 12 h of virtual simulation using i-Human cases each week for 8 weeks during the COVID-19 pandemic. Group 1 and Group 2 participated in pre-conference and post-conference (debriefing) for each clinical or virtual simulation experience.	Maternal–pediatric course	No statistically significant difference was observed between the traditional and virtual learning groups.
Bode, S. et al., 2021 Germany [19]	6 pediatric nursing students	2 week–week rotation on the Interprofessional Training Ward in Pediatrics (IPAPAED) was replaced by the Interprofessional COVID-19 Replacement Program (I-reCovEr) in four 60-min face-to-face sessions.	Interprofessional training ward in pediatrics	No differences were observed.
Bradford, H. et al., 2021 Columbia [20]	Midwifery and Women’s Health Nurse Practitioner	It used synchronous and asynchronous simulations for formative learning. A majority of students accessed one or more of these simulations: Adapted simulation opportunities: objective structured clinical examinations (synchronous), IUD—intrauterine device training (synchronous), trigger films (synchronous or asynchronous), bilateral learning tools (asynchronous), and suturing skills simulations (synchronous).	Women’s health nursing	These simulation opportunities serve as valuable adjuncts to traditional learning and provide a levelling of experience to students with variable accessibility and capability to engage in the clinical setting. Some virtual opportunities may be implemented before entry to the clinical setting to promote skill acquisition, use of person-centered language, and student confidence. Simulated clinical experiences are an evidence-based approach for developing and enhancing the acquisition of clinical and communication skills, decision-making, and self-confidence. Provides readiness to begin or return to the clinical setting.
Cowperthwait, A., et al., 2021 USA [21]	80 senior undergraduate students	Clinical practice was replaced by simulation.	Psychiatric mental health	It was valued as a reflective pause in the middle of the simulation was possible; students learned by watching other colleagues interact with the same patient; the ability to discuss following responses or important lines; receiving feedback.
Fung, J. et al., 2021 Hong Kong [22]	188 final-year nursing undergraduate students	A virtual simulation education program with debriefing replaces the traditional clinical practicum in the COVID-19 situation.	Medical and surgical cases	A significant improvement was perceived by students in clinical competence and the nursing process. Self-efficacy has also been boosted. Communication and critical thinking were applied better in the traditional clinical environment.
Hassler, L. et al., 2021 USA [23]	98 s-degree nursing students and 11 clinical groups	Flipped clinical practice: synchronized remote clinical experience to simulate the experience of the traditional hospital setting	Students had to choose one clinical specialty: adult health, mental health, pediatrics or obstetrics.	The flipped clinical experience was a successful methodology to reinforce clinical concepts.
Hwang, H. & Chun, Y., 2021 Republic of Korea [24]	59 randomly expressed nursing students: (*n* = 30) experimental group; (*n* = 29) control group	Clinical practice education using virtual reality. The experimental group used the vSim of a nursing program, and the control group of nursing students did not use the vSim of a nursing program as an alternative practice for clinical practice.		Applying and not applying simulation clinical practice education using virtual reality positively affected critical thinking disposition and clinical practice performance, but it was not statistically significant.
Kim, M., Kang, H. & Gagne, J., 2021 Republic of Korea [25]	20 nursing students	Use of virtual simulation as an alternative to clinical practice for nursing with six steps: (a) suggested reading, (b) pre-simulation quizzes that provide students with an overview of the contents, (c) interactive clinical nursing scenarios authorized by the NLN, (d) post-simulation quizzes, (e) documentation assignments, and (f) guided reflection questions.	“Unspecified information”	Difficulties were encountered in using the virtual simulation because students needed to use English, which was not their native language and some specific cultural differences; Benefits to student confidence and competence in providing patient-centered care: it allowed the user to care for patients from admission to discharge by themselves, and they were able to self-assess and strengthen their skills through repeated questionnaires, a feedback log, and reflection. Gaps in satisfaction due to a need for improvement: some students reported a lack of reality and the limited function of the vs. and stated that the vs. differs fundamentally from reality. The care is given by pressing buttons rather than by communicating directly with, and providing nursing care to, patients, thus allowing certain essential activities to be ignored.
Revell, S. et al., 2022 USA [26]	93 undergraduate nurse students	Traditional clinical hours were supplemented with 18 h of on-campus simulation experiences, 6 self-paced case studies, participation in COVID-19 vaccination and/or testing clinic activities, and two 2-h synchronous online seminars. The students developed 2 scholarly journals focused on reflection and application of knowledge to clinical practice as well as a self-reflection paper.	Medical–surgical	Transformative learning was evident in the writing of the students. Students demonstrated response to change, discovering resilience, developing confidence, finding gratitude, embracing advocacy, and transforming and becoming a nurse. Students recognized the opportunities mentorship afforded them, despite challenges.
Shea, K. & Rovera, E., 2020 USA [27]	244 nursing students	Using virtual simulations and remote simulations as telehealth with standardized patients provided an alternative for 50% of the required direct patient care hours during the COVID-19 pandemic and campus closure.	Nursing Fundamentals and Community Health Promotion and Wellness Reproductive Health and Mental Health Medical/Surgical and Pediatrics Advanced Medical/Surgical and Community Health	The inability to complete the required clinical hours can delay the graduation dates of some students, disrupting the new nurses entering the workforce. Finding ways to replace clinical practice hours with simulation activities has become a priority.
Wands, L., Geller, D., & Hallman, M., 2020 USA [28]	42 senior nursing students	Over 4 weeks, students collectively logged over 1200 h of simulation time, attending approximately 100 sessions. Students used 4 free online simulation programs to substitute in-person clinical experiences: - Canadian Alliance of Nurse Educators Using Simulation (CAN-Sim). High-quality video-based virtual simulations focus on adult acute care scenarios involving medical diagnoses of urosepsis, diabetic ketoacidosis, and respiratory distress; The Virtual Healthcare Experience with an opportunity to explore a virtual hospital with five different departments: emergency, pediatrics, medical–surgical, maternal and child, and mental; National League for Nursing’s (NLN) Advancing Care Excellence Series in the form of clinical scenarios with six vulnerable populations: pediatrics, veterans, seniors, individuals with disabilities, Alzheimer’s patients, and caregivers of individuals with Alzheimer’s; Augmented Reality Integrated Simulation Education (ARISE) included simulation scenarios containing real-life storylines with four levels that increase in complexity from basic assessment to crisis. Scenarios cover the topics of chest pain, heart failure, wound management, pediatric asthma, obstetrics, therapeutic communication, and end-of-life.	“Unspecified information”	The students reported experiencing positive growth in confidence in their assessment skills, ability to prioritise care and interventions, communication with patients and their families and the health care team, and providing interventions that foster patient safety. Less positive aspects included difficulties encountered when trying to manage multiple technological devices to display videos or other materials from websites, sessions being cancelled on short notice, and the inability to ensure student engagement if the student did not turn or keep their camera on

## Data Availability

Not applicable.
